# The Tissue-Engineered Human Psoriatic Skin Substitute: A Valuable In Vitro Model to Identify Genes with Altered Expression in Lesional Psoriasis

**DOI:** 10.3390/ijms19102923

**Published:** 2018-09-26

**Authors:** Geneviève Rioux, Claudia Pouliot-Bérubé, Mélissa Simard, Manel Benhassine, Jacques Soucy, Sylvain L. Guérin, Roxane Pouliot

**Affiliations:** 1 Centre LOEX de l’Université Laval, Génie Tissulaire et Régénération, Centre de Recherche FRQS du CHU de Québec, Axe Médecine Régénératrice, Québec, QC G1J 1Z4, Canada; genevieve.rioux.9@ulaval.ca (G.R.); claudia.pouliot-berube.1@ulaval.ca (C.P.-B.); melissa.simard.6@ulaval.ca (M.S.); 2 Faculté de Pharmacie, Université Laval, Québec, QC G1V 0A6, Canada; 3 Centre Universitaire d’Ophtalmologie-Recherche, Centre de Recherche FRQS du CHU de Québec, Axe Médecine Régénératrice, Québec, QC G1S4L8, Canada; manal.benhassine.1@ulaval.ca (M.B.); sylvain.guerin@fmed.ulaval.ca (S.L.G); 4 Département d’Ophtalmologie, Université Laval, Québec, QC G1V 0A6, Canada; 5 Département de Dermatologie, Hôpital de l’Enfant-Jésus, Québec, QC G1J 1Z4, Canada; jacques.soucy@fmed.ulaval.ca

**Keywords:** psoriasis, gene profiling, tissue-engineering

## Abstract

Psoriasis is a chronic inflammatory skin disease for which no cure has emerged. Its complex etiology requires the development of an in vitro model representative of the pathology. In this study, we exploited gene profiling analyses on microarray in order to characterize and further optimize the production of a human psoriatic skin model representative of this in vivo skin disease. Various skin substitutes were produced by tissue-engineering using biopsies from normal, healthy donors, or from lesional or non-lesional skin samples from patients with psoriasis, and their gene expression profiles were examined by DNA microarray. We demonstrated that more than 3540 and 1088 genes (two-fold change) were deregulated between healthy/lesional and lesional/non-lesional psoriatic substitutes, respectively. Moreover, several genes related to lipid metabolism, such as *PLA2G4E* and *PLA2G4C*, were identified as repressed in the lesional substitutes. In conclusion, gene profiling analyses identified a list of deregulated candidate genes associated with various metabolic pathways that may contribute to the progression of psoriasis.

## 1. Introduction

Psoriasis is a chronic skin affection characterized by well-demarcated red scaly plaques that appear most of the time on elbows, knees, the sacroiliac region, nails, and the scalp [1,2]. The disease affects both men and women in the same proportion, and can be classified into two subtypes, according to the onset of the disease [3,4]. Plaque psoriasis is the most common, but other forms, such as erythrodermic psoriasis, inverse, pustular, and guttate, are frequently observed [5,6,7]. The disease has a different incidence depending on ethnicity and geographical distribution [8]. The latest statistics indicate that 7.4 million people in the United States, and 125 million individuals worldwide are affected by psoriasis [9]. The physical consequences of plaque psoriasis are more important than the simple fact that they provide pain and pruritus. Indeed, the impacts on general health go beyond what we previously believed as there is a substantial list of psoriasis-associated comorbidities, including Crohn’s disease, psoriatic arthritis, atherogenic dyslipidemia, hypertension, diabetes, and several more [10]. The psychological consequences are worth mentioning as many patients will suffer from anxiety and depression [11].

Despite being an incurable disease, several treatments have emerged. They are essentially based on the deep knowledge we have about immunology and genetics, the two main components of the pathogenesis that makes psoriasis a very heterogeneous disease [12,13]. In this sense, the quest for the miracle drug to treat psoriasis is not a simple task. In addition, a better psoriasis model could help reduce the huge costs associated with the development of new drugs aimed at reducing the skin lesions caused by this disease [14]. Then, a more accurate model for an early stage of preclinical research would perhaps eliminate molecules earlier in the developing process. Indeed, research in this area is hampered by a lack of a model that adequately represents the pathology. The models used for psoriasis research can be classified into two broad, well-described categories: in vivo and in vitro [15,16]. In vivo models, that mostly exploit murine models, are divided into three sub-categories: spontaneous, genetically engineered, and xenotransplantation [17,18]. These models have several strengths and weaknesses, but none accurately represent human skin. In vitro models, in turn, can be valuable research tools to study specific pathways [19]. The general classification for in vitro psoriatic models relies on whether they represent 2D or 3D reconstructed skin substitutes. The monolayer model (2D) was largely used despite the fact that no interactions can be observed between the different cell subtypes that normally constitute the human skin, which also caused an increased interest in the 3D model [20]. Our team has been working for several years on a reconstructed human psoriatic skin that uses fibroblasts and keratinocytes from psoriatic plaques [21]. Our psoriatic skin model has been extensively characterized for its biochemical and immunohistochemical properties [22,23,24]. However, the genetic component, very important in the pathology of this disease, has not yet been explored. The present study was aimed at comparing the pattern of genes expressed in our model of human psoriatic lesional skin with that of healthy and non-lesional skin substitutes produced using the same technique.

## 2. Results

### 2.1. Macroscopic and Histological Analyses

**Figure 1:**
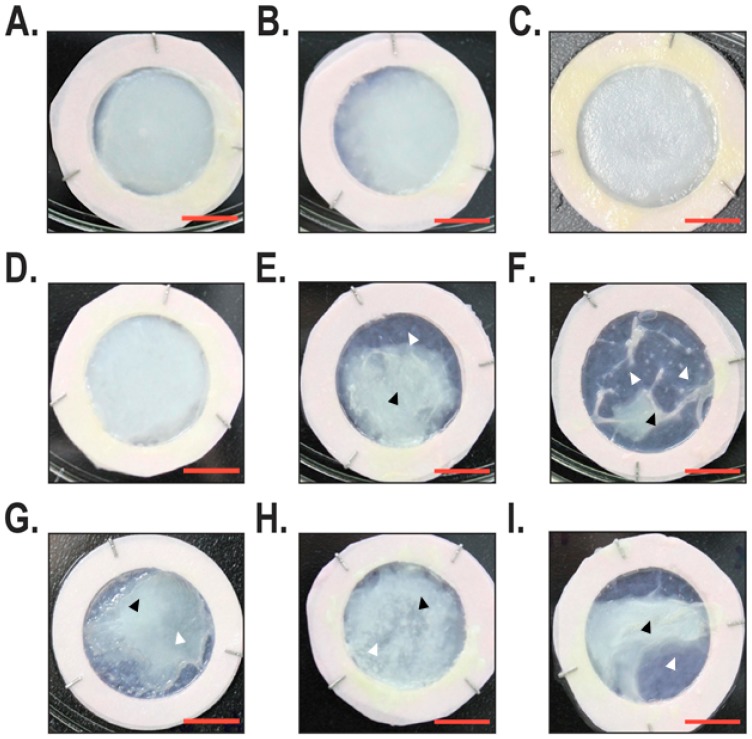
Macroscopic analysis of the reconstructed skin substitutes. For each group, tissue-engineered skin substitutes were produced with three different cell populations. (**A**–**C**) Tissue-engineered skin substitutes produced with healthy fibroblasts and keratinocytes. (**D**–I) Tissue-engineered skin substitutes produced with fibroblasts and keratinocytes isolated from either non-lesional (**D**–**F**) or lesional (**G**–**I**) psoriatic skin. Scale bar = 1 cm. Black arrowheads indicate the position of protuberant regions, whereas white arrowheads position thinner regions of the reconstructed skin substitutes.

An examination of reconstructed skin substitutes under phase contrast microscopy showed that the healthy substitutes (Figure 1A–C) all had the same macroscopic aspect without any differences related to cell populations. Indeed, the appearance of the skin substitutes was regular and uniform. The macroscopic aspect of the non-lesional substitute showed two different phenotypes, one closer to the healthy skin substitutes (Figure 1D) and the other being more similar to the lesional skin substitutes (Figure 1E,F). Skin substitutes reconstructed with lesional cells isolated from psoriatic plaques were very similar to one another, all being irregular with thinner and thicker skin areas (Figure 1G–I).

**Figure 2:**
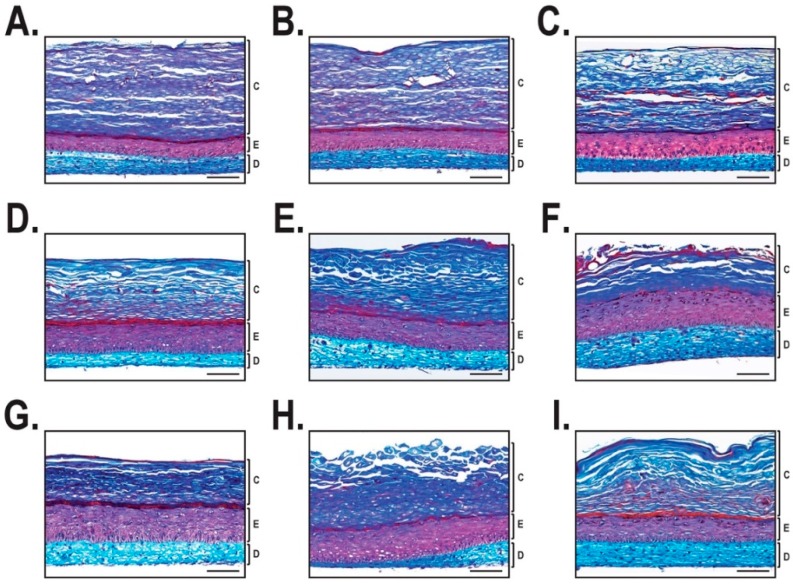
Histological analysis of the reconstructed skin substitutes. Mason’s trichrome staining after 21 days of culture at the air-liquid interface. For each group, tissue-engineered skin substitutes were produced with three different cell populations. (**A**–**C**) Healthy skin substitutes. (**D**–**F**) Non-lesional psoriatic skin substitutes. (**G**–**I**) Lesional psoriatic skin substitutes. Scale bar = 100 µm. C: Stratum corneum; E: Epidermis; D: Dermis.

The histological appearance of healthy skin substitutes reconstructed from different human cell populations appeared similar to one another (Figure 2A–C). Indeed, the thickness of the dermis (in blue) did not show any significant difference, and the epidermis (in purple) maintained a uniform thickness. On the other hand, the non-lesional skin substitutes had a thicker epidermis than that seen for healthy substitutes (Figure 2D–F). The lesional skin substitutes demonstrated a thicker epidermis than the healthy skin substitutes and showed a more disorganized structure (Figure 2G–I).

### 2.2. Gene Profiling Analysis of the Most Deregulated Genes between Healthy, Lesional and Non-Lesional Skin Substitutes

**Figure 3:**
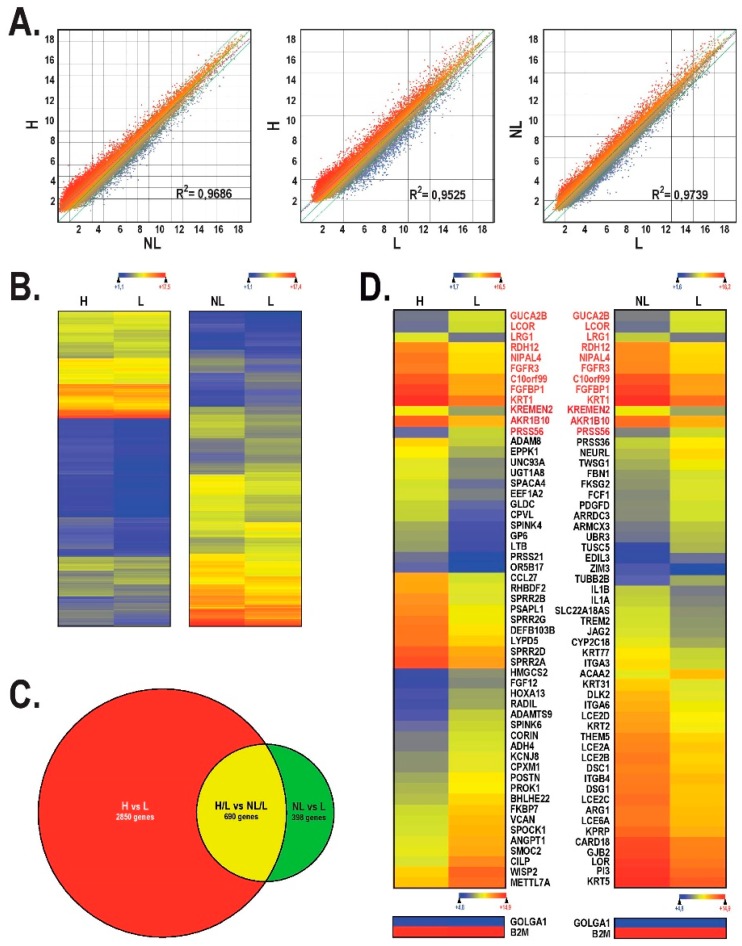
Microarray analysis. (**A**) Scatter plot of log2 of signal intensity from 60,000 different targets covering the entire human transcriptome of healthy or non-lesional tissue-engineered skin substitutes (in the *y*-axis) against non-lesional (first graph) or lesional (last two graphs) (in the *x*-axis). (**B**) Heatmap representation of genes whose expression is differentially regulated by at least two-fold in healthy against lesional substitutes and in non-lesional against lesional substitutes. (**C**) Venn diagram that indicates the number of deregulated genes in healthy against lesional substitutes (red circle) and in non-lesional against lesional substitutes (green circle). Genes that are commonly deregulated between these two groups are indicated in yellow. (**D**) Heatmap representation of the 55 most deregulated genes expressed by lesional substitutes relative to healthy substitutes (left) and non-lesional substitutes against lesional substitutes (right). The most highly expressed genes are shown in red, while the most weakly expressed are in blue. The genes with red writing are those identified as similarly deregulated between the two heatmaps. H: healthy; L: lesional; NL: non-lesional.

We next conducted a gene profiling analysis on the microarray in order to compare the gene expression profile between healthy, lesional, and non-lesional skin substitutes. The scatter plot analysis of the 60,000 probes loaded on the chip showed important changes in the pattern of genes expressed by the lesional (L) skin substitutes against the healthy (H) skin substitutes (Figure 3A, middle panel; R^2^ = 0.9525). The comparison between the healthy and the non-lesional (NL) skin substitutes revealed that the number of genes deregulated between these two conditions decreased (Figure 3A, left panel; R^2^ = 0.9686). Very much the same was observed when deregulated genes from both the lesional and non-lesional skin substitutes were analyzed against each other (Figure 3A, right panel; R^2^ = 0.9739).

A heatmap representation for all the genes showing a two-fold or more expression variation unique to the non-lesional or lesional substitutes relative to the healthy substitutes was generated (Figure 3B). An analysis of the heatmap from the healthy against the lesional condition indicates that 3540 candidate genes were deregulated by more than two-fold (Figure 3B, left; Figure 3C). The number of genes that fit into this category dropped to 1088 (69% reduction) when non-lesional and lesional conditions were compared to each other (Figure 3B, right; Figure 3C).

We next analyzed the data files from the microarray to sort out genes whose expression was the most greatly deregulated between the lesional and either the non-lesional or healthy conditions (Figure 3D). The *Arraystar* program was used to restrict the search to the 55 most deregulated genes in the healthy against lesional condition and in the non-lesional against lesional condition. However, in order to avoid inter-individual variations that may occur in the pattern of expressed genes (see Figure S1), data shown in Figure 3D (left and right columns) were collected from the comparison between pools of reconstructed skin substitutes produced from two healthy cell populations and from lesional and non-lesional reconstructed skin substitutes produced from three different cell populations. Among these highly deregulated genes, 12 (*GUCA2B*, *LCOR*, *LRG1*, *RDH12*, *NIPAL4*, *FGFR3*, *C10orf99*, *FGFBP1*, *KRT1*, *KREMEN2*, *AKR1B10*, and *PRSS56*) were similarly deregulated when comparing the healthy and lesional, and the non-lesional and lesional, conditions (genes in red on Figure 3D). 

### 2.3. Alteration in Cytokines, Chemokines and Growth Factors Gene Expression in Healthy vs. Lesional Skin Substitutes

As the immune system is expected to have a significant impact on the pattern of genes expressed by the pathologic skin of patients with psoriasis, we therefore searched our gene profiling data files to sort out cytokines, chemokines, growth factors, and some of their receptors encoding genes whose expression is altered between healthy and lesional skin substitutes. From the 257 cytokine/growth factor genes present on the array, only 14 had their expression altered by more than two-fold (Table 1), with the remaining genes being either not expressed or having their expression level unaltered in both conditions (healthy vs. lesional). Among the affected genes, *IL1R2*, *CXCL13*, *CXCL14*, *EPO*, *INHBA*, and *TNFSF9* had their expression increased between 2.1 and 6.8-fold, whereas the expression of *IL15*, *CXCL2*, *CCL20*, *CXCL1*, *LTB*, *TNFRSF10A*, *IL24*, and *CCL27* was reduced by a factor of 2.1 to 6.6-fold in lesional compared with healthy skin substitutes. Expression of the gene encoding cathelicidin antimicrobial peptide (*CAMP*), whose expression has been reported to increase in psoriasis [25], was also found to increase by 2.4-fold in our lesional skin substitutes (Table 1).

### 2.4. Gene Ontology Analysis

Gene ontology (GO) assignments were conducted on the 55 most deregulated genes (Figure 3D), using the amigo gene ontology database, to illustrate which biological processes are altered between the different conditions (Table 2) [26]. The most deregulated pathways in the healthy against the lesional condition include ‘keratinization’ (*p* = 3.625 × 10^−4^), ‘isoprenoid metabolic process’ (*p* = 5.922 × 10^−4^), and ‘retinoid metabolic process’ (*p* = 2.321 × 10^−3^). The products encoded by the most strongly deregulated genes between the non-lesional against the lesional condition are particularly involved in ‘skin development’ (*p* = 1.151 × 10^−11^), ‘keratinization’ (*p* = 7.952 × 10^−6^), and ‘epidermis development’ (*p* = 1.088 × 10^−5^). A closer and more extended examination of the genes identified as deregulated in our lesional substitutes within each of these biological processes is presented in Table S1. Of the 28 genes selected within these biological functions and identified as deregulated in psoriasis in both the present study and that reported by Gudjonsson et al. [27], a total of 19 (68%) were similarly deregulated in both analyses, therefore further validating the reliability of our model.

The results presented in Table 3 illustrate the differences (both the linear signals and fold-changes are indicated) in the expression of genes involved in lipid synthesis and the establishment of the skin barrier. The conditions compared are: healthy and non-lesional substitutes (H vs. NL), healthy and lesional substitutes (H vs. L), and non-lesional and lesional substitutes (NL vs. L). Several genes related to collagen expression were more strongly deregulated between healthy and lesional substitutes (H vs. NL). Also, several enzymes related to lipid metabolism such as phospholipases, protein kinases, and lipoprotein lipase had their expression altered in the lesional (H vs. KL) and non-lesional (H vs. NL) substitutes compared with healthy ones.

## 3. Discussion

Despite the fact that the cellular and immunohistological aspects of our in vitro psoriatic model were largely characterized [21,24,28], they missed the detailed transcriptomic analyses that could help verify the integrity of our reconstructed psoriatic skin. To fulfill this need, we produced a large number of healthy and both lesional and non-lesional psoriatic substitutes in order to demonstrate whether the progression of this disease might result from alterations in the pattern of genes expressed by the cells from these reconstructed tissues. Both macroscopic and immunohistochemical analyses revealed that the healthy substitutes presented in Figure 1A–C have a very similar phenotype to one another. A well-differentiated stratum corneum that contributes to maintaining an appropriate barrier function is also observed. Previous studies have determined that the lipid domain of the stratum corneum from healthy substitutes was comparable to that found in the in vivo healthy skin [28].

Unlike healthy substitutes, psoriatic substitutes made from non-lesional cells presented phenotypic variations between the different cell populations tested (Figure 1D–F). First, we observed that one of them (Figure 1D) had a phenotype similar to that observed with the healthy substitutes, which means that the appearance was regular and opaque. Nonetheless, others (such as Figure 1E,F) appeared similar to the psoriatic substitutes by their irregular and contracted appearance. As a result, two different profiles could be proposed for these substitutes: one related to the healthy and the other to the psoriatic phenotype. These results support previous research conducted in our laboratory where two different expression profiles were confirmed by histological, immunohistochemical, and macroscopic analyses for substitutes reconstructed using non-lesional psoriatic cells [22]. Finally, the macroscopic and histological data support the psoriatic phenotype of our lesional psoriatic substitutes, as also described in previous work [21]. However, it is important to point out that the histological features of the cell population used in Figure 2I appeared to be less pronounced than the other two cell populations used for the reconstruction of lesional psoriatic skin substitutes. Indeed, several studies on psoriasis characterized it as a cyclic disease, with periods of remission and exacerbation of plaques, meaning that the characteristics of the plaques change over time, sometimes appearing to be exacerbated or healing [29,30]. The state wherein the psoriatic plaque was at the time of the biopsy may be an intrinsic component of the cells used to produce the reconstructed psoriatic substitutes.

A recent review by Niehues et al. has listed the most complete and best-validated models that include major psoriasis hallmarks with regard to the gene and protein expression profile and epidermal morphology [31]. Our model appears to be the only one that fulfills the complete expression signatures related to acanthosis, parakeratosis, and hyperproliferation. Although tissue-engineered, 3D models are structures that advantageously represent the in vivo condition, the fact remains that the in vitro results are not necessarily the perfect mirror of the in vivo observations [32]. Therefore, to extensively characterize and validate the psoriasis-like features of the 3D models, Niehues et al. suggested the use of not just one marker, but rather of a panel of disease-associated genes and proteins for the validation of novel psoriasis models [31].

The data files from the gene profiling studies were first examined using scatter plot analyses and have provided particularly interesting details on important changes occurring in the pattern of genes expressed in our three studied conditions (healthy, non-lesional, and lesional). A total of five cell populations were pooled to represent the healthy condition (H), whereas both the lesional (L) and non-lesional (NL) conditions were each constituted of three and four pooled cell populations, respectively. Three scatter plots have been generated from these data. First, the graph of the healthy against the lesional condition identified a significant amount of deregulated genes, which was supported by the slope of the linear regression of 0.9525. Thereafter, the graph with the most reduced number of deregulated genes was that which compared the non-lesional against the lesional condition, with an R^2^ value of 0.9739. Based on these two scatter plots, it can be concluded that there are many deregulated genes between healthy and lesional substitutes, but much less between non-lesional and lesional cells from the same patient. This graph is particularly important since the comparison that is made here did not take into account the inter-individual differences since the samples (lesional and non-lesional) came from the same four patients. Moreover, one of the most important points to emerge from this study was the scatter plot comparing the healthy against the non-lesional substitutes. The latter had a linear regression slope of 0.9686, demonstrating that the healthy skin of an individual who has never been affected by psoriasis is different from healthy skin of a patient that suffers from psoriasis. To our knowledge, there are no non-lesional psoriatic skin models available on the market to which we can refer in order to establish any possible correlation with our results, despite the fact that the non-lesional skin has been increasingly characterized [33,34]. In regard to the in vivo skin, in all studies encountered, morphological and histological characteristics were similar between the healthy skin of a patient that had never suffered from psoriasis and the healthy skin of a psoriatic patient. Nevertheless, a few studies have reported that many genes, whose encoded protein products are associated with the processes of angiogenesis and lymphogenesis, are overexpressed in non-lesional skin [35,36]. Our gene profiling data also show that despite similar histological characteristics, non-lesional psoriatic cells demonstrate a disruption in their gene expression pattern that is less important than the one observed for lesional tissues, but more important than the healthy one.

The Venn diagram analysis was then used to define the number of deregulated genes specific to each experimental condition. An examination of the diagram revealed that a large number of genes are expressed in very different ways between healthy and lesional substitutes. Indeed, of the previous 3540 genes identified as deregulated between healthy and lesional substitutes, 2850 genes were found to be specific to that condition (healthy vs. lesional), whereas 690 genes were also found to be deregulated between non-lesional and lesional substitutes. In the same sense, 398 genes out of the 1088 genes previously identified for non-lesional and lesional substitutes were also found to be specific to this condition (non-lesional vs. lesional). It was no surprise to note a greater number of deregulated genes between healthy and lesional than non-lesional and lesional substitutes. Interestingly, and based on these results, we can assume that the 398 genes specifically deregulated between the non-lesional and lesional conditions most likely account for the progression of the non-lesional state to the lesional one.

The type of array used and the nature of the samples, for instance, tissue-engineered psoriatic skin substitutes, make our work even more innovative, but also very difficult to compare to those published in the literature. Indeed, several groups have studied the pattern of genes expressed by lesional and non-lesional skin in vivo [33,34]. Among them, Gudjonsson et al. identified an impressive amount of deregulated genes between psoriatic and healthy skin (1326 genes), while 1085 genes were identified as deregulated between lesional and non-lesional skin [27]. Oestreicher et al. also generated a complete list of 159 genes that define psoriasis in molecular terms and whose expression was compared between lesional and non-lesional psoriatic skin [34]. These data are in accordance with those presented in the present study. Indeed, from the 41 randomly selected genes among those identified as deregulated in psoriasis by Gudjonsson et al. and Oestreicher et al., a total of 22 (54%) were found to be similarly deregulated (repressed or activated) in our psoriatic skin substitutes, therefore demonstrating the reliability of our in vitro model (Table S2). In spite of this, the difference in the expression we observed for some of these genes in regard to other studies may rely on differences such as the type of array used and the nature of the samples. It is important to stress that the RNAs extracted directly from biopsies do not go through the same steps as those extracted from our reconstructed tissues. Indeed, and unlike RNA extracted from biopsies, the cells of the reconstructed psoriatic tissues from which RNA was extracted were exposed to additional stresses that can also impact the pattern of expressed genes. Our reconstructed psoriatic skin only contains keratinocytes and fibroblasts, whereas a skin biopsy contains all the different cell types from the skin. Some cell types may have a gene expression profile that stands out in the dataset obtained in the ex vivo studies, whereas the profile in our study only involves skin fibroblasts and keratinocytes. This aspect may also explain the difference observed between the two studies.

The mystery of whether the primary abnormality of psoriasis resides in epidermal keratinocytes or dermal immunocytes remains unsolved. Conventional wisdom portrays psoriasis as an autoimmune disease, where misguided T lymphocyte activities cause secondary epithelial abnormalities despite the fact that a few researchers believe that psoriasis is a genetically determined, abnormal epithelial response to infectious and/or physicochemical skin insults [37]. There is a cumulative body of evidence that the psoriatic epidermis has both structural and functional abnormalities [38]. It is therefore plausible that psoriatic keratinocytes tease the immune system, waking it from its slumber to secrete a barrage of cytokines and factors in a misguided attempt to restore keratinocytes to their normal proliferative state. Consistent with this hypothesis, we observed variations in the expression of a few cytokines or growth factors by our lesional skin substitutes, despite the fact that they are devoid of any immune cells, suggesting that their expression is likely keratinocyte-specific. In agreement with the results generated using a mouse model of skin psoriasis [39], a near seven-fold increase in the expression of the *CXCL13* chemokine was observed in our lesional skin substitutes relative to the level observed in the healthy substitutes. It is also particularly interesting to point out that the near seven-fold repression we observed for *CCL27* gene expression in our lesional substitutes was also reported in punch biopsies from human psoriatic lesions [40]. Consequently, our in vitro model can, indeed, be used to identify genes with altered expression in psoriasis, and maybe new, yet unidentified genes as well, such as *CXCL2*, *CXCL14*, *INHBA*, *EPO*, *TNFSF9*, and *IL1R2*. Therefore, our results confirmed and extended previous studies highlighting the remarkable potential of keratinocytes to produce pro-inflammatory cytokines, adhesion molecules, growth factors, and chemotactic polypeptides [23].

Several research teams around the world are working on the gene expression of healthy, non-lesional and lesional skin, allowing us to compare our in vitro model to theirs. A few studies reported that increased gene expression in psoriatic skin is partly attributable to keratinocytes, T lymphocytes, and macrophages, but also to stimulation by pro-inflammatory cytokines [41,42]. In contrast, the repression of some specific genes in the psoriatic plaques would be mediated by subcutaneous adipose tissues and the epidermis [43]. These findings were made possible through ontological analyses that assign genes differentially expressed to biological processes or cell types. Since only two cell types are present in our model (fibroblasts and keratinocytes), an analysis of the biological processes has been achieved using the 55 most deregulated genes identified in our study. Among these biological activities, the keratinization process was greatly altered across all the deregulated genes, as well as that of isoprenoid metabolism and retinoids in lesional and healthy substitutes. As for non-lesional and lesional substitutes, the most altered biological processes were more related to skin and epidermis development. Although our model is not entirely complete as it lacks vascularization, innervation, and immune cells, it nevertheless demonstrates similarities with the metabolic pathways affected in psoriasis [2]. So far, there is no doubt that the keratinization process is greatly affected in psoriasis, also leading to disturbances in the organization and composition of lipids of various epidermal layers, thus explaining the ontology results [44,45]. In contrast to the various lipid changes orchestrated into the psoriatic plaque itself, healthy skin areas in psoriatic patients seem to have very little lipid changes [46].

Table 3 helped identify several key players in the lipid metabolism of the stratum corneum and skin in general. These genes are repressed in the lesional psoriatic substitutes compared to the healthy substitutes. Indeed, the phospholipase A2 epsilon and gamma genes (*PLA2G4E* and *PLA2G4C*) were downregulated by 3.7- and 3.9-fold, respectively. Type A2 phospholipase catalyzes the hydrolysis of glycerophospholipids, thereby releasing arachidonic acid and lysophospholipids [47]. It is interesting to note that lysophospholipids are mediators of cell communication that can affect various cellular processes, including proliferation/apoptosis and cellular remodeling [48]. Specifically, phospholipase A2 epsilon (PLA2G4E) is involved in the input mechanisms of the cell, or the clathrin-independent mechanism of tubule formation involved in compound transportation to the cell surface [49]. The phospholipase A2 gamma (encoded by the gene *PLA2G4C*) participates in the constitutive release of various lipids, including arachidonic acid, oleic acid, and some fatty acids [50]. The phospholipase A2 gamma also plays a role in oxidative stress mechanisms in order to fix lipid damage [51]. Psoriasis is known and recognized as having disturbances in lipid, as well as oxidant-antioxidant, metabolism [52,53]. These imbalances may be linked to the products of genes for which expression is altered, such as is the case with *PLA2G4E* and *PLA2G4C*. Unfortunately, no direct link has been reported between the expression of these genes and the imbalances mentioned above. The gene for dystonin (*DST*), a member of the plakines family associated with adhesion and cellular junction through intermediate filament, was significantly down-regulated between healthy and involved substitutes (four-fold change) [54]. No direct link has been mentioned for any modification of its expression in psoriasis; however one of the isoforms causes the expression of a protein called BPAG1-e (bullous pemphigoid antigen 1), which is found in bullous pemphigoid skin disease [55]. This protein would affect both the migration and adhesion of keratinocytes [56]. In addition, besides its interaction with both clathrin and actin, dystonin participates in the formation of the hemidesmosomes through its interaction with the β4 integrin subunit [57,58]. Interestingly, a few studies reported that some patients with psoriasis also developed bullous dermatosis spontaneously or following medical treatments [59,60,61], thereby suggesting that a relationship between the autoimmune disease of bullous pemphigoid and psoriasis exists.

Detailed analysis of the genes shown in Figure 3D allowed us to make multiple comparisons with a number of genes associated with the late cornified envelope family, as is the case for *LCE2A*, *LCE2B*, *LCE2C*, *LCE2D*, and *LCE6A*. Indeed, these genes were down-regulated in lesional compared to non-lesional substitutes. It is particularly interesting to point out that the protein products of these genes are associated with the stratification of the epidermis. Consistent with our observations, deletion within the *LCE3B* and *LCE3C* genes (*LCE3B_LCE3C*) was shown to be associated with an increased risk of developing psoriasis [62,63]. Similarly, genes associated with loricrin (*LOR*), various keratins (*KRT1*, *KRT2*, *KRT5*, *KRT31*, and *KRT77*), and interleukins (*IL1A* and *IL1B*) were also found to be strongly deregulated in our human psoriatic skin model compared to the reconstructed non-lesional skin [64,65].

Recent findings have established the skin as a peripheral neuroendocrine organ that is tightly networked to central stress axes. This capability contributes to the maintenance of the skin’s and body’s homeostasis. Specifically, epidermal and dermal cells produce and respond to classical stress neurotransmitters, neuropeptides, and hormones, and this production is modified by ultraviolet radiation and biological, chemical, and physical factors [66,67,68]. Therefore, skin removed from the natural neuro-immuno-endocrine connections in the body is certainly deprived of the local neuroendocrine capabilities that skin can normally compensate for. Although this withdrawal from central communication may affect the results of our research, it remains interesting to think that in view of local neuro-endocrine-cytokine activities, organ culture models can provide clinically useful information [66,68,69,70,71]. For instance, it has been demonstrated that the corticotrophin-releasing factor, the pro-opiomelanocortin, and corresponding receptors were co-expressed in cultured keratinocytes, melanocytes, or dermal fibroblasts [72,73]. Moreover, the expression of the executive arm of the cutaneous hypothalamo-pituitary adrenal axis, i.e., the production of cortisol and corticosterone, has been clearly demonstrated in cultured epidermal keratinocytes and melanocytes, as well as in dermal fibroblasts [73,74,75,76,77]. Nevertheless, it is important to stress that the model described in our study, even if more complete than cultured cells grown as monolayers, still remains a simplified representation of one of the most important and complex organs of the human body: the skin.

In conclusion, transcriptome analysis of our tissue-engineered lesional and non-lesional substitutes allowed us to establish a list of the most deregulated genes whose encoded products can contribute to the induction and/or the progression of this disease. Moreover, we have been able to associate these genes with altered biological processes and make correlations with other pathologies. The changes in the expression noted for the genes herein identified may have resulted from processes within the cell itself, but also from feedback activation mechanisms and repression in various metabolic pathways. Plaque psoriasis is a disease for which causes seem to be multifactorial, making the creation of a unique gene profile implausible. The more there will be analyses of gene expression, methylation status, and post-transcriptional processes, the clearer the explanatory picture of the disease will be.

## 4. Materials and Methods

This study was conducted in agreement with the Helsinki declaration and was performed under the guidelines of the research ethics committee of the ‘CHU de Québec’ (ethic code: DR-002-1387, protocol renewal approved on 6 June 2018). All patients were given adequate information to provide written consents.

### 4.1. Cell Culture

Fibroblasts were cultured in Dulbecco–Vogt modification of Eagle’s medium (DMEM) supplemented with 10% bovine growth serum (HyClone^®^, Thermo Fisher Scientific, Ottawa, ON, Canada), 100 UI/mL penicillin G (Sigma, Oakville, ON, Canada), and 25 µg/mL gentamicin (Schering, Pointe-Claire, QC, Canada). Keratinocytes were cultured in a combination of DMEM with Ham’s F12 (3:1), supplemented with 5% Fetal Clone II serum (Hyclone), 5 µg/mL insulin (Sigma), 0.4 µg/mL hydrocortisone (Calbiochem, EMD Biosciences, Gibbstown, NJ, USA), 10^−10^ M cholera toxin (MP Biomedicals, Montreal, QC, Canada), 10 ng/mL human epidermal growth factor (EGF; Austral Biological, San Ramon, CA, USA), 100 UI/mL penicillin G (Sigma), and 25 µg/mL gentamicin (Schering).

### 4.2. Production of Tissue-Engineered Substitutes

All skin substitutes (healthy, lesional, and non-lesional) were produced using the self-assembly method with some modifications [21,78]. Different substitutes were produced using nine different cell populations; five from healthy patients (18-, 22-, 23-, 38-, and 46-year old) and four from psoriatic patients (47-, 49-, 64-, and 70-year old). The psoriatic cells were used to produce lesional and non-lesional substitutes. Briefly, fibroblasts (passage 6) were cultured in the presence of ascorbic acid (Sigma) at a concentration of 50 µg/mL. After 28 days of culture, these cells formed sheets that could be superimposed and incubated further for seven days to form a new dermal layer before keratinocytes (passage 2) were seeded on top of it to form a new epidermal layer. After one week of submerged culture in DMEM-HAM, the substitutes were raised to the air–liquid interface. Biopsies of skin substitutes were taken after 21 days of culture at the air–liquid interface and analyzed by gene profiling on DNA microarrays [79].

### 4.3. Gene Expression Profiling

Total RNA was isolated from five different healthy (18-, 22-, 23-, 38-, and 46-year old) and eight different psoriatic reconstructed skin substitutes (four lesional and four non-lesional (47-, 49-, 64-, and 70-year old)) using the RNeasy Mini Kit (QIAGEN, Toronto, ON, Canada). Cyanine 3-CTP labeled cRNA targets were prepared from 50 ng of total RNA using the Agilent One-Color Microarray-Based Gene Expression Analysis kit (Agilent Technologies, Santa-Clara, CA, USA). Then, 600 ng cRNA was incubated on a G4851A SurePrint G3 Human GE 8 × 60 K array slide (60,000 probes, Agilent Technologies). Slides were then hybridized and scanned on an Agilent SureScan Scanner. Data were analyzed using ArrayStar V4.1 (DNASTAR, Madison, WI, USA) software for the generation of heat maps and scatter plots. Biological replicates were as follows: for the experiment conducted on the healthy tissue-engineered skin, total RNA was obtained from two different reconstructed skin substitutes produced using keratinocytes and fibroblasts cultured from the skin of two different healthy donors (22- and 46-year old); for the experiments that used reconstructed psoriatic skin substitutes, RNA was obtained from four different reconstructed non-lesional (NL) and lesional (L) skin substitutes produced using keratinocytes and fibroblasts cultured from the skin of four different psoriatic donors (47-, 49-, 64-, and 70-year old). All data generated from the arrays were also analyzed by robust multiarray analysis (RMA) for background correction of the raw values. They were then transformed in Log2 base and quantile normalized before a linear model was fitted to the normalized data to obtain an expression measure for each probe set on each array. All microarray data presented in this study comply with the Minimum Information about a Microarray Experiment (MIAME) requirements (GEO # (GSE120464, http://www.ncbi.nlm.nih.gov/geo/query/acc.cgi?acc=GSE120464)).

### 4.4. Histological Analysis

Three biopsies of each reconstructed tissue combination were fixed with HistoChoice’s solution and embedded in paraffin wax. Five-micrometer-thick sections were cut and stained with Masson’s Trichrome (Zeiss, Axio Imager, North York, ON, Canada).

## Figures and Tables

**Table 1:** Deregulated cytokines and growth factors.

Gene Symbol	Gene Name	Linear Signal		Fold Change (L/H)
		Healthy (H)	Lesional (L)	
*IL1R2*	Interleukin-1 receptor type 2, soluble form	21.111	52.487	2.486 up
*CXCL13*	C-X-C motif chemokine 13	8.956	60.844	6.793 up
*CXCL14*	C-X-C motif chemokine 14	5475.355	26,650.308	4.867 up
*CAMP*	Cathelicidin antimicrobial peptide	26.904	65.127	2.416 up
*EPO*	Erythropoietin	23.081	94.42	4.090 up
*INHBA*	Inhibin beta A chain	132.158	293.07	2.217 up
*TNFSF9*	Tumor necrosis factor ligand superfamily member 9	110.858	332.436	2.998 up
*CXCL2*	C-X-C motif chemokine 2	334.366	158.747	2.106 down
*CCL20*	C-C motif chemokine 20	135.671	48.3	2.808 down
*CXCL1*	Growth-regulated alpha protein	1739.554	656.318	2.650 down
*LTB*	Lymphotoxin-beta	57.051	16.161	3.530 down
*TNFRSF10A*	Tumor necrosis factor receptor superfamily member 10A	179.232	84.848	2.112 down
*IL24*	Interleukin-24	164.625	67.68	2.432 down
*CCL27*	C-C motif chemokine 27	4802.716	731.651	6.564 down
*IL15*	Interleukin-15	368.781	174.554	2.112 down

**Table 2:** Gene ontology analysis.

Pathways	Sample Frequency (*n*)	*p*-Value
Healthy against lesional condition		
Keratinization (GO: 0031424)	4	3.625 × 10^−4^
Isoprenoid metabolic process (GO: 0006720)	5	5.922 × 10^−4^
Retinoid metabolic process (GO: 0001523)	4	2.321 × 10^−3^
Diterpenoid metabolic process (GO: 0016101)	4	3.077 × 10^−3^
Biological process (GO: 0008150)	50	4.007 × 10^−3^
Keratinocyte differentiation (GO: 0030216)	4	5.312 × 10^−3^
Non-lesional against lesional condition		
Skin development (GO: 0043588)	13	1.151 × 10^−11^
Keratinization (GO: 0031424)	5	7.952 × 10^−6^
Epidermis development (GO: 0008544)	8	1.088 × 10^−5^
Single-organism developmental process (GO: 0044767)	27	2.897 × 10^−5^
Organ development (GO: 0048513)	20	2.984 × 10^−5^
Developmental process (GO: 0032502)	27	3.619 × 10^−5^

**Table 3:** Selection of genes whose expression is altered through the conditions.

Gene	Protein Name	Linear Signals			Fold Change		
		H	NL	L	H vs. NL	H vs. L	NL vs. L
*AREG*	Amphiregulin	4556.114	3649.748	1740.864	1.248 down	2.617 down	2.096 down
*CCL27*	C-C motif chemokine 27	4802.716	1196.792	327.991	4.012 down	14.646 down	3.649 down
*CERS3*	Ceramide synthase 3	1205.298	1076.517	545.522	1.119 down	2.209 down	1.973 down
*COL10A1*	Collagen alpha-1(X) chain, Collagen type X alpha 1	40.35	153.551	223.581	3.805 up	5.540 up	1.456 up
*COL4A1*	Collagen alpha-1(IV) chain	624.384	1325.645	1377.716	2.123 up	2.206 up	1.039 up
*COL4A2*	Collagen type IV alpha 2, Collagen alpha-2(IV) chain	3008.066	7008.3	7538.532	2.329 up	2.506 up	1.075 up
*COL5A3*	Collagen alpha-3(V) chain	81.195	201.437	272.105	2.480 up	3.351 up	1.350 up
*COL6A3*	Collagen alpha-3(VI) chain, Uncharacterized protein	216.945	264.225	666.849	1.217 up	3.073 up	2.523 up
*COL8A1*	Collagen alpha-1(VIII) chain	179.866	373.282	419.755	2.075 up	2.333 up	1.124 up
*COL9A3*	Collagen alpha-3(IX) chain; Collagen, type IX, alpha 3	672.004	561.211	177.532	1.197 down	3.785 down	3.161 down
*CXCL10*	C-X-C motif chemokine 10	18.223	16.471	6.664	1.106 down	2.734 down	2.471 down
*DMRTA1*	Doublesex- and mab-3-related transcription factor A1	149.171	72.609	29.644	2.054 down	5.032 down	2.449 down
*DST*	Dystonin	2666.323	2193.123	654.854	1.215 down	4.071 down	3.349 down
*FABP6*	Gastrotropin	396.131	270.887	105.903	1.462 down	3.740 down	2.557 down
*LPL*	Lipoprotein lipase	354.755	560.341	1138.306	1.579 up	3.208 up	2.031 up
*NOD2*	Nucleotide-binding oligomerization domain-containing protein 2	1002.491	893.358	445.888	1.122 down	2.248 down	2.003 down
*PIK3R2*	Phosphatidylinositol 3-kinase regulatory subunit beta	2620.755	5460.444	6354.303	2.083 up	2.424 up	1.163 up
*PLA2G4C*	Cytosolic phospholipase A2 gamma	287.492	73.724	73.553	3.899 down	3.908 down	1.002 down
*PLA2G4E*	Cytosolic phospholipase A2 epsilon	260.291	205.142	71.176	1.268 down	3.657 down	2.882 down
*PNPLA5*	Patatin-like phospholipase domain-containing protein 5	15.78	81.347	98.806	5.155 up	6.261 up	1.214 up
*POSTN*	Periostin	85.28	478.01	1044.007	5.605 up	12.242 up	2.184 up

## Supplementary Materials

Supplementary materials can be found at http://www.mdpi.com/1422-0067/19/10/2923/s1.
