# Supplementary material for: The Tissue-Engineered Human Psoriatic Skin Substitute: A Valuable In Vitro Model to Identify Genes with Altered Expression in Lesional Psoriasis
Source: Int J Mol Sci. 2018 Sep 26;19(10):2923. doi: 10.3390/ijms19102923 (PMC6213003; doi:10.3390/ijms19102923)
Supplement: Supplementary file 1 [file ijms-19-02923-s001.pdf]

Supplementary Materials: The Tissue-Engineered Human Psoriatic Skin Substitute: A Valuable In Vitro Model to Identify Genes with Altered Expression in Lesional Psoriasis

Geneviève Rioux, Claudia Pouliot-Bérubé, Mélissa Simard, Manel Benhassine, Jacques Soucy, Sylvain L. Guérin and Roxane Pouliot *

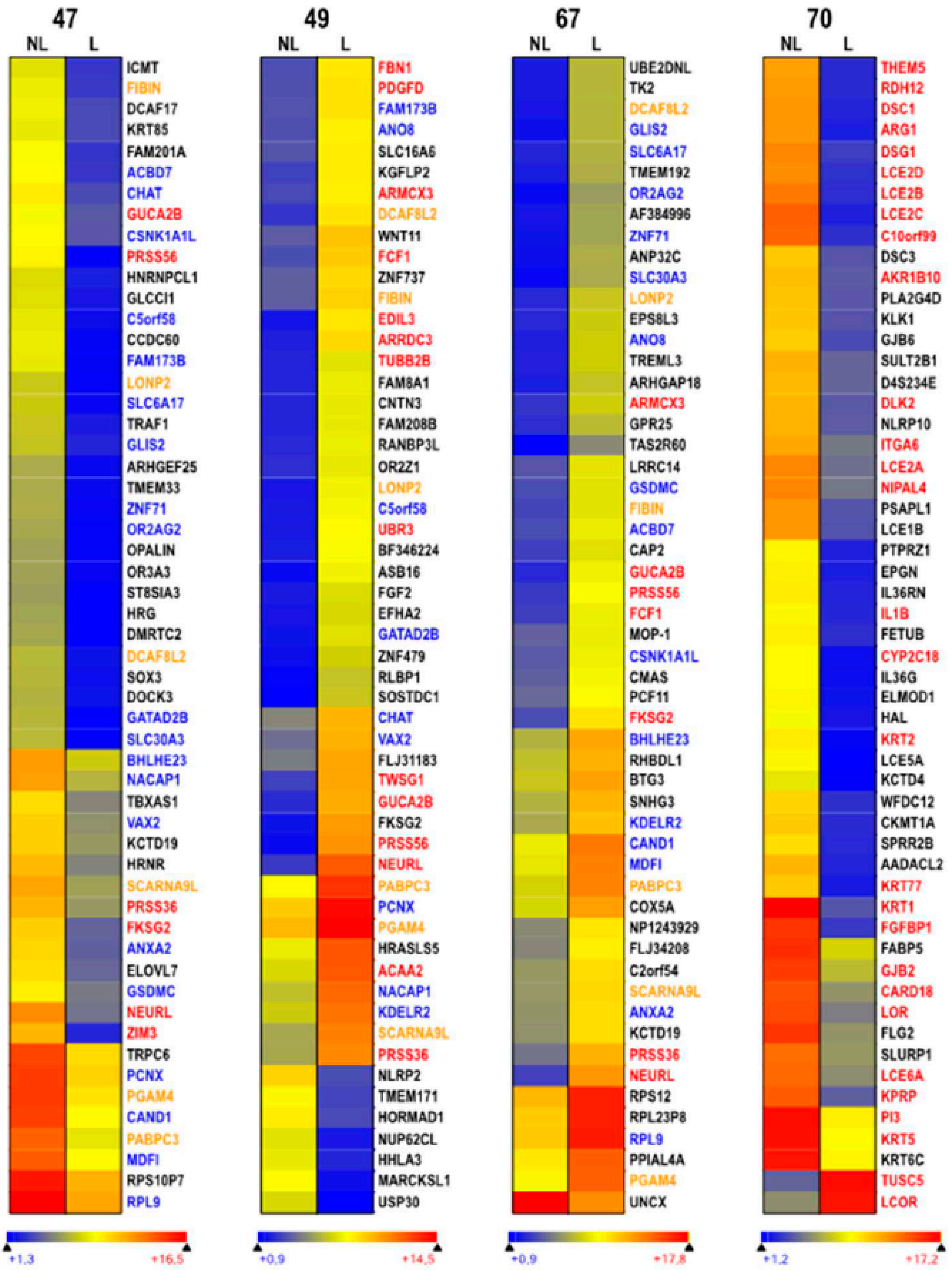

Figure S1. Gene profiling analysis of the most deregulated genes in non-lesional vs lesional psoriatic substitutes. Heatmap representation of the 55 most deregulated genes expressed by non-lesional (NL) against lesional (L) substitutes produced using both fibroblasts and keratocytes from four different cell populations of psoriatic patients (47-, 49-, 64-, and 70-year old). Genes in red have also been identified in the 50 most deregulated genes from the pooled data files shown in Figure 3D. Those indicated in orange and blue are deregulated in three- and two- out of the four individual substitutes, respectively.

Table S1. Distribution of psoriasis-related deregulated genes within biological processes.

Biological Process	Genes Symbol
Regulation of T-cell cytokine production (GO:0002724)	*HMOX1, IL1B, ARG1*
Positive regulation of chemokine production (GO:0032722)	*ALOX15B, HMOX1, IL1B, POSTN*
Cornification (GO:0070268)	*SPRR3, KRT2, KRT15, KRT1, LOR, SPRR2B, SPRR2G, PI3*
Keratinization, keratinocyte differentiation and epidermal cell differentiation (GO:0031424, GO:0030216 and GO:0009913)	*SPRR3, KRT2, LCE1B, KRT15, SPRR4, KRT1, LOR, SPRR2B, SPRR2G, PI3*
Skin developpement (GO:0043588)	*SPRR3, KRT2, LCE1B, KRT15, SPRR4, FLG2, KRT1, LOR, SPRR2B, SPRR2G, PI3, LTB*
Acute inflammatory response (GO:0002526)	*IL1B, SERPINA3*
Regulation of angiogenesis (GO:0045765)	*HMOX1, IL1B, CXCL13, KRT1, FGFBP1*
Regulation of cell migration (GO:0030334)	*ALOX15B, HMOX1, IL1B, CXCL13, POSTN, PDGFD, FGFBP1, C10orf99*
Myeloid leukocyte mediated immunity (GO :0002444)	*AMPD3, SERPINB9, SERPINA3, CST6, FLG2, KRT1, ARG1*
Immune system process (GO:0002376)	*ATP1B1, HMOX1, IL1B, AQP9, CCL27, AMPD3, SERPINB9, CXCL13, SERPINA3, CST6, FLG2, KRT1, ARG1, LTB, PI3, C10orf99*
Response to wounding (0009611)	*SPRR3, HMOX1, ARG1, POSTN*
Immune response (GO:0006955)	*IL1B, AQP9, CCL27, AMPD3, SERPINB9, CXCL13, SERPINA3, CST6, FLG2, KRT1, ARG1, LTB, PI3*

Genes whose name are indicated in black are similarly deregulated (either repressed or activated), whereas those in red are regulated in opposite ways between our study and that of Gudjonsson et al. [1].

Table S2. Comparison between published microarray datasets with our most deregulated genes (L vs H and L vs NL).

Gene Symbol	Gene Name	Gudjonsson et al. [1]		Oestreicher et al. [2]	Our Study	
		L vs NL fold change	L vs H fold change	L vs NL fold change	L vs NL fold change	L vs H fold change
KRT15	Keratin, type I cytoskeletal 15	0.406	0.405		0.380	0.377
C10orf99	Putative uncharacterized protein C10orf99	22.862	41.394		0.159	0.204
SERPINA12	Serpin A12	0.206	0.242		0.359	0.358
SERPINA3	Alpha-1-antichymotrypsin; Serpin peptidase inhibitor, member 3	2.429	2.222		1.243	3.530
SERPINB9	Serpin B9	2.604	2.098		1.682	2.304
CXCL13	C-X-C motif chemokine 13	5.900	6.005		1.435	8.034
AQP9	Aquaporin-9	0.252	0.306		0.360	0.402
ALOX15B	Arachidonate 15-lipoxygenase B	0.826	0.485		0.294	0.579
AMPD3	AMP deaminase 3	1.954	2.232		1.449	1.968
FGFBP1	Fibroblast growth factor-binding protein 1	3.142	3.567		0.089	0.111
LCE1B	Late cornified envelope protein 1B	0.500	0.595		0.293	0.548

KRT1	Keratin, type II cytoskeletal 1	0.126	0.131		0.123	0.141
ACADL	Long-chain specific acyl-CoA dehydrogenase, mitochondrial	0.485	0.462		0.501	0.653
AKR1B10	Aldo-keto reductase family 1 member B10	40.647	57.643		0.231	0.109
FLG2	Filaggrin-2	0.328	0.349		0.349	0.387
UNC93A	Protein unc-93 homolog A	2.134	2.710		0.460	0.124
GLDC	Glycine dehydrogenase [decarboxylating], mitochondrial	0.579	0.406		0.162	0.669
LTB	Lymphotoxin-beta	1.901	2.087		0.374	0.217
CCL27	C-C motif chemokine 27	0.117	0.129		0.274	0.068
SPRR2B	Small proline-rich protein 2B	20.232	35.983		0.296	0.075
SPRR2G	Small proline-rich protein 2G	4.733	8.063		0.253	0.068
SPRR3	Small proline-rich protein 3	4.659	6.614		1.199	6.223
SPRR4	Small proline-rich protein 4	0.469	0.373		0.519	0.728
LYPD5	Ly6/PLAUR domain-containing protein 5	1.783	2.101		0.207	0.293
HMGCS2	Hydroxymethylglutaryl-CoA synthase, mitochondrial	0.457	0.444		3.341	19.263
POSTN	Periostin	0.424	0.385		2.184	12.242
CILP	Cartilage intermediate layer protein 1	0.409	0.371		2.526	6.396
WISP2	WNT1-inducible-signaling pathway protein 2, WISP2 protein	0.576	0.463		2.289	1.843
PDGFD	Platelet-derived growth factor D	0.551	0.475		4.446	1.725
EDIL3	EGF-like repeat and discoidin I-like domain-containing protein 3	0.485	0.465		4.741	2.637
IL1B	Interleukin-1 beta	2.159	2.051		0.207	0.490
CYP2C18	Cytochrome P450 2C18	1.904	2.148		0.233	0.292
ARG1	Arginase-1	2.651	3.665		0.208	0.317
GJB2	Gap junction beta-2 protein	6.945	7.311		0.225	0.245
LOR	Loricrin	0.447	0.486		0.201	0.479
PI3	Elafin	93.902	130.746		0.230	0.306
CST6	Cystatin-M	0.356	0.343	0.32	0.445	0.712
HMOX1	Heme oxygenase 1	2.110	2.174	2.5	1.878	2.503
ATP1B1	Sodium/potassium-transporting ATPase subunit beta-1			3.2	4.607	1.673
TUBB2	Tubulin beta-2A chain			2.6	4.535	1.062
KRT2	Keratin, type I cytoskeletal 20			0.37	0.170	0.092

Genes whose name are indicated in black are similarly deregulated (either repressed or activated), whereas those in red are regulated in opposite ways between our study and those of Gudjonsson et al. [1] and Oestreicher et al. [2].

References

1. Gudjonsson, J.E.; Ding, J.; Johnston, A.; Tejasvi, T.; Guzman, A.M.; Nair, R.P.; Voorhees, J.J.; Abecasis, G.R.; Elder, J.T. Assessment of the psoriatic transcriptome in a large sample: Additional regulated genes and comparisons with in vitro models. *J. Invest. Dermatol.* **2010,** *130*, 1829-1840.

2. Oestreicher, J.L.; Walters, I.B.; Kikuchi, T.; Gilleaudeau, P.; Surette, J.; Schwertschlag, U.; Dorner, A.J.; Kruger, J.G.; Trepicchio, W.L. Molecular classification of psoriasis disease-associated genes through pharmacogenomic expression profiling. *Pharmacogenomics J.* **2001**, *1*, 272-287.
